# Challenges in Feeding Children with Autism Spectrum Disorder: the Role of Nurses in Research and Interventions

**DOI:** 10.17533/udea.iee.v42n3e01

**Published:** 2024-10-11

**Authors:** Márcio Flávio Moura de Araújo

**Affiliations:** 1 RN, PhD. Oswaldo Cruz Foundation: Eusébio, Ceará, Brazil. E-mail: marcio.moura@fiocruz.br. https://orcid.org/0000-0001-8832-8323 Fundação Oswaldo Cruz Oswaldo Cruz Foundation: Eusébio Ceará Brazil marcio.moura@fiocruz.br

The prevalence of autism spectrum disorder (ASD) has increased significantly in every continent in the world. Research findings suggest that nearly 1 in every 100 children is diagnosed with ASD globally.^1^ In the United States, more recent data from the Centers for Disease Control and Prevention (CDC) indicate an even higher prevalence, with 1 in every 36 children, or close to 2.0% being diagnosed with the disorder.^2^ (Zeiddan *et al.,* 2022; CDC, 2023). The prevalence distribution varies among different regions. In Latin American countries, like Brazil, data is limited, but it is estimated that approximately 1.5- to 2-million children could be affected by ASD. In Africa, prevalence rates are lower, with significant variations among countries due to differences in data collection methods and the diagnostics resources available​.^3^^,^^4^

In this regard, it has become quite common for nurses to provide health care to children with ASD and their respective families in the most diverse health services and territories. I would like to draw attention to nurses' challenges when implementing food and nutrition-based interventions and/or research in these cases. Initially, it is important for nurses to consider that these children may have problems, such as eating disorders. In case of feeding problems, in ASD, that refers to selective or avoidant feeding behaviors that do not involve cognitive concern with bodily image, weight, or shape. That is, selective eating can occur (picky eating can affect 91% of these children), along with refusal of food, avoidance of certain types of food, and idiosyncratic food preferences (such as insistence on certain foods or forms of presentation). In cases of eating disorders in ASD, it is possible to highlight persistent eating disturbances or food-related behaviors that negatively impact the individual's health or functioning, often associated with excessive concern about weight, shape, and bodily image.^5^ Hence, nurses must face, above all, food selectivity, as a predictor or aggravating factor of problems in nutritional status from malnutrition to overweight. Both, impacting negatively on the child growth and development. Nursing interventions must also integrate the families of these children, given that family stress related with meals is overwhelming. Parents and caregivers of children with ASD frequently report difficulties in managing the child’s feeding preferences and guarantee adequate nutrition. That stress can create a tense environment during meals, further harming the quality of food.^6^ In addition, children with ASD are more prone to developing dysbiosis, a condition that can be exacerbated by poor diet, like excessive consumption of ultra-processed foods, which is on the rise throughout the world.^7^

In the academic field, nurses play a fundamental role in conducting research on nutrition, from topics like breastfeeding, enteral and parenteral nutrition, to research issues such as nutrition in children with ASD, especially in primary care.^3^^,^^8^^,^^9^ However, they are likely to confront a number of obstacles. One of the principal challenges is the lack of instruments specifically validated to evaluate the feeding habits and behaviors of children with ASD. The majority of the questionnaires and scales available to study feeding habits do not take into account the peculiarities of said population, hindering the collection of accurate data.

Another important research niche that nurses need to unveil is the limited understanding of the relationship between family eating habits and the symptomatologic characteristics of ASD. Depending on the autism spectrum level, food can be an aggravating or alleviating factor for symptoms, and that is often neglected in academic studies.^10^ In this case, the possibility exists of observational studies, mixed methods studies, and even qualitative studies. In the latter case, nurses can explore how dimensions of family health are impacted during this stressful process of feeding a child with ASD, researching aspects such as maternal burnout, support networks, and issues of food insecurity.

Nurses around the world can also be very successful when researching nursing diagnoses, such as those proposed by the North American Nursing Diagnosis Association (NANDA), in children with ASD. Especially those related to the class "Imbalanced Nutrition: Less Than Body Needs". Studies conducted with this focus, in clinical practice and in research centers, can elucidate the early prevalence of nursing diagnoses, such as "Risk of metabolism for unstable blood glucose level"; “Dynamics of ineffective child feeding”, and “Risk of excess weight” in primary care services. This favors not only early interventions, but also damage reduction, health promotion actions, advocacy, and inclusion of the child-family binomial in the respective health services.^11^

Nurses inclined toward translational or clinical research may focus attention on how increased intake of ultra-processed foods affects ASD symptomatology and contributes to dysbiosis. The answers to these questions can go beyond the clinical sphere, going against aspects directly linked to social health determinants, like food insecurity, public policies, and training the nursing staff on the topic of autism. Urging nurses to intervene and research the nutrition of children with ASD provides ammunition against the war that seeks to rob the future of this increasing number of children with ASD globally. With effort, nurses will be able to shed light on practical and scientifically based solutions for this scenario; going through the development of instruments, well-designed nursing diagnoses, and translational research. 
